# Statistical and Clinical Report of Three Hundred Consecutive Cases of Labor

**Published:** 1890-08

**Authors:** Henry C. Buswell

**Affiliations:** 358 South Division Street; Buffalo, N. Y.


### STATISTICAL AND CLINICAL REPORT OF THREE HUNDRED CONSECUTIVE CASES OF LABOR.[1]

By HENRY C. BUSWELL. M. D., Buffalo, N. Y.

The following report embraces my obstetrical work since I began practice, about two years ago:

### PRESENTATIONS.

Single deliveries: Occipito-læva-anterior, 217, or 72⅓ per cent.; occipito-dextra-anterior, 21, or 7 per cent.; occipito-dextra-posterior, beginning and terminating as such, 7, or 2⅓ per cent.; occipito dextra-posterior, changing to occipito-dextra-anterior, 14, or 4⅔ per cent.; occipito-læva-posterior, 1, or ⅓ of 1 per cent.; breech, 8 cases, or 2⅔ per cent.; vertex (exact presentation not determined), 28 cases, or 9⅓ per cent.

Multiple deliveries: Case 64, primipara, age 26: First child, sacro-dextra-anterior; second child, occipito-læva-anterior. A single placenta with two cords. The amniotic sac was divided by a membranous partition, so that each child had a separate sac.

Case 91, multipara, age 32: First child, occipito-læva-anterior; second child, occipito-dextra-anterior. Each child contained in separate sac, with distinct and separate placentæ. Father's sister, mother, and grandmother had all borne twins.

Case 126, primipara, age 36: Membranes of first child ruptured on Monday evening, but child not born until Saturday morning. First child, occipito-dextra-anterior; second child, occipito-læva-anterior. Long forceps to deliver both. Single placenta with double sac; placenta adherent.

Case 180, multipara, age 27: First child, occipito-læva-anterior; second child, occipito-dextra-anterior. Weight: boy, 9 pounds 4 ounces; girl, 8 pounds. Large double placenta and sac.

Totals: Primiparæ, 96; multiparæ, 204. Children born alive, 287; dead, 13. Sex of single deliveries: males, 170; females, 126. Sex of multiple deliveries: males, 2; females, 6.

---

1. Read before the Buffalo Obstetrical Society, June 24, 1890.

### BREECH PRESENTATIONS.

Primiparæ, 4; multiparæ, 4. In six cases the presentation was sacro-dextra-anterior; in one sacro-læva-anterior; and in one the presentation was not determined.

Four children were born alive; two were dead and macerated when born; one was born dead at the eighth month; one, before referred to as the case in which the presentation was not determined, was attended by a midwife, and when seen by me the body of the child was born and child dead. Forceps were applied and head extracted, the occiput being anterior.

In case of the presentation sacro-læva-anterior, the mother had had seven previous deliveries, several with the use of forceps, and only three living children. I applied forceps as soon as the body was delivered, but quite an amount of force was used in extracting the head, and the child was dead.

### FORCEPS.

I have used the forceps forty-six times, or once in six and one-half cases. Short forceps, twenty-four times; long forceps, twenty-two times; seven times in medium operations, and fifteen times in high operations.

Case 121, primipara, 26: Called at three A. M.; applied forceps at seven P. M. After one hour and no progress, Dr. Lothrop was called in consultation. He was unable to lock Tarnier's forceps, but applied White's, and after powerful traction long continued, a dead baby was delivered. Bi-parietal diameter of baby's head, measured with pelvimeter, was four and one-eighth inches.

Case 129, multipara: Applied long forceps, on account of prolapse of cord, but child born dead.

Case 197, multipara: Has had several dead-born children, said to have been of unusual size. Slow labor; applied short forceps and easily delivered head, but could not extract shoulders until the child was dead. Largest child ever seen by me; was unable to obtain weight, but length was twenty-two inches.

### ADHERENT PLACENTÆ.

Case 121, primipara; 36: Twins; long forceps.
Case 170, primipara; 29; no forceps.
Case 243, multipara; 25; short forçeps.
Case 277, multipara; 31; short forceps.

My custom is to wait until the patient complains of pain before attempting to deliver the placenta, which is accomplished by expression.

In none of the cases given above did I introduce my hand into the uterus under one hour from the birth of the child.

### PLACENTA PREVIA.

Case 146, multipara, 40: When first seen, the woman was collapsed and pulseless, as she had been flowing at intervals for a week. Whisky was used hypodermically. Os dilated to the size of a silver quarter, with placenta previa centralis; child presenting occipito-læva-anterior. Turning was easily accomplished by external manipulation; os was then forcibly dilated to size of a silver dollar, and the hand introduced between the placenta and uterus until the membranes were reached, which were ruptured and a foot seized and brought down. The child was delivered almost at once, with considerable traction, and was dead. The uterus contracted well, with no hemorrhage. The patient was conscious and able to speak, but in fifteen minutes had an anemic convulsion and died.

I regret the haste with which this delivery was accomplished.

### SEPTICEMIA.

Case 18, primipara: Was not seen after the third day; her condition was normal until the ninth day, when septicemia existed. Uterus was curetted and washed out by Dr. Banta, but not much *dêbris* obtained. Recovery after forty-two days of constant attendance.

Case 25, primipara: Chill on fifth day. Curetted by Dr. Banta, with negative results. Recovery in two weeks. I think both of these cases would have had a fatal termination had it not been for Dr. Banta's timely aid, and had I had another case at that time I should have given up obstetrics in despair.

Case 296, multipara, 26: Chill on morning of third day. Curette and douche used at once. Some placental tissue and a small bunch of membranes removed. Recovery in eight days.

Three of my cases have been followed by pelvic cellulitis, and had a tardy recovery.

### HEMORRHAGES.

Case 81, primipara, 18: Two hours after birth of child pulse 150 per minute. Uterus dilated and filled with clotted blood.

Cases 163 and 207 were complicated with post-partum hemorrhage, and are of no especial interest except that I was taught by them that hot intrauterine injections would not stop hemorrhage unless the womb was first emptied of clots.

Case 143, primipara, 32: March 2, 1889, Dr. Banta saw this patient, who had been taken with sudden flooding, which had ceased upon his arrival. There was no dilatation of the os and the doctor did nothing but send word to me that it was a probable case of placenta previa, and requested me to remain at the office during the day in order that I might go to the patient quickly. Saw her at five P. M., and found large quantity of blood in the bed.

Emptied vagina and found no dilatation of os. Exposed os and could see that there was no blood escaping from it, yet the lower blade of the speculum soon became filled with blood. Found a small artery upon anterior wall of vagina, midway between clitoris and cervical attachment of vagina, ruptured and spurting. This was ligated, and there was no further trouble. April 18th, the patient was delivered of a good-sized infant presenting O. L. A., and the placental attachment was at or near the fundus. There was no history of injury.

Case 276, Xpara: When first seen, patient had lost a large amount of blood, was still flowing, and os the size of a ten cent piece; tamponed and waited two hours, during which time there was renewal of labor pains. Sent for Dr. Banta, and upon his arrival removed the tampon and found os sufficiently dilated for application of forceps, which was done by Dr. Banta, to the head lying in the transverse diameter of the superior strait. Child born dead, and the mother had a protracted recovery.

### ALBUMINURIA.

I have seen eight cases of albuminuria in pregnant women; six have been complicated with convulsions.

I induced premature labor in one case at the seventh month and had a living child. The usual remedies had been tried without avail.

Another case was seen not five minutes before the birth of the child; the mother had been ill and under the care of another physician for several weeks; the rhetinitis of albuminuria had caused blindness. The patient died within twenty-four hours after delivery.

### ANOMALIES.

Case 269, multipara: Embolism of anterior tibial artery with large slough two months and one week previous to labor.

Case 254, primipara, 19: Labor of twenty-four hours; twelve hours afterward, pulse 130; temperature, 105; no chill; subsidence of bad symptoms within twenty-four hours.

### CASES OCCURRING IN THE PRACTICE OF DR. R. L. BANTA, IN WHICH I HAVE RENDERED ASSISTANCE WITH THE PRIVILEGE OF CAREFUL EXAMINATION.

Placenta previa centralis: Child presented O. L. A. Turning by external manipulation; dead child; good recovery of mother.

The following three cases of dystocia were consecutive labors in the same patient:

(1.) Scapulo-dextra-anterior; turning by the bi-polar method; dead child. It was recognized that there was a diminished antero-posterior diameter of superior strait.

(2.) Sacro-dextra-anterior: Head extracted with great difficulty and delay, and only by application of forceps.

(3.) Foot presentation; rectification by external manipulation; Tarnier's forceps applied to head in O. L. A. position; forceps slipped repeatedly; uterus ruptured; child extracted with great difficulty even then; death of mother within one hour.

Primipara: Mento-læva-anterior presentation changed by Banta's method to occipito-dextra-anterior; forceps then applied to fix the head in the pelvic canal, and the case was terminated by nature.

Multipara: Was seen by me on November 20th, when a diagnosis of transverse presentation, with head to the right, was made. December 16th she was attended by Dr. Banta, who verified the diagnosis; turned and delivered.

Multipara: Previous labors always instrumental. High operation; head could not be made to engage even by repeated and forcible traction with White's forceps, but with less than one-eighth of the traction force previously used the head easily engaged with the Tarnier forceps.

Primipara: Protracted labor of forty-eight hours without engagement of breech, which presented sacro-dextra-anterior. Tarnier's forceps applied to breech and delivery accomplished in less than one hour. No mark of the blades could be found upon the body of the child, but, unfortunately, the cord was severed by one of the blades.

OBSERVATIONS IN CONCLUSION.

In multiparæ with uterine inertia, I have administered ergot in five drop doses every fifteen minutes until good uterine contractions.

In primiparæ, morphine has been used to facilitate dilatation and lessen pain.

For after-pains, ergot has been used frequently.

Mortality: Of my own cases, *nil.* Counting the cases of placenta previa and albuminuria, occurring in the practice of other physicians, two-thirds of one per cent.

358 South Division Street.

